# Antibody-powered nucleic acid release using a DNA-based nanomachine

**DOI:** 10.1038/ncomms15150

**Published:** 2017-05-08

**Authors:** Simona Ranallo, Carl Prévost-Tremblay, Andrea Idili, Alexis Vallée-Bélisle, Francesco Ricci

**Affiliations:** 1 Department of Chemistry, University of Rome, Tor Vergata, Via della Ricerca Scientifica, 00133 Rome, Italy; 2 Laboratory of Biosensors and Nanomachines, Département de Chimie, Départment de Biochimie et Médecine Moléculaire, Université de Montréal, Montréal, Quèbec, Canada H3T 1J4

## Abstract

A wide range of molecular devices with nanoscale dimensions have been recently designed to perform a variety of functions in response to specific molecular inputs. Only limited examples, however, utilize antibodies as regulatory inputs. In response to this, here we report the rational design of a modular DNA-based nanomachine that can reversibly load and release a molecular cargo on binding to a specific antibody. We show here that, by using three different antigens (including one relevant to HIV), it is possible to design different DNA nanomachines regulated by their targeting antibody in a rapid, versatile and highly specific manner. The antibody-powered DNA nanomachines we have developed here may thus be useful in applications like controlled drug-release, point-of-care diagnostics and *in vivo* imaging.

## Abstract (web summary)

Responsive molecular machines can perform specific tasks triggered by environmental or chemical stimuli. Here, the authors show that antibodies can be used as inputs to modulate the binding of a molecular cargo to a designed DNA-based nanomachine, with potential applications in diagnostics and drug delivery.

One of the most exciting research paths in the field of nanotechnology and supramolecular chemistry is aimed at rationally designing and developing responsive molecular machines that, like naturally occurring proteins, can perform a specific function in response to a certain molecular input^1^,^2^,^3^,^4^,^5^. Several supramolecular nanodevices of increasing chemical complexity have been described in the recent years for applications ranging from controlled release of a therapeutic cargo^6^, signal transduction^7^,^8^ and sensing^9^.

With its highly predictable base-pairings, its low cost, ease of synthesis and biocompatibility, DNA has become the material of choice to design and engineer nanomechanical devices and machines that display specific structures and functions^10^,^11^,^12^,^13^,^14^. A wide range of DNA-based nanodevices have been reported that, in response to a specific molecular cue, can give a signal, release a cargo or perform a directional motion^15^,^16^,^17^,^18^,^19^,^20^,^21^,^22^. Despite their impressive performances, a limitation affects these DNA-based nanodevices: their activity, in fact, is usually triggered by a quite restricted class of molecular cues and inputs. These inputs range from environmental stimuli (like pH or temperature)^23^,^24^ to chemical inputs that, in the majority of cases, are limited to DNA strands or, more seldom, to small molecules and proteins^25^,^26^,^27^. Several DNA-based sensors, able to signal the presence of a specific antibody, have been reported to date^28^,^29^,^30^. However, the demonstration of DNA nanomachines performing more complex functions (such as, for example, drug-release) and employing antibodies as regulatory inputs has witnessed only limited efforts^31^,^32^.

Thus motivated, here we report an antibody-powered DNA-based nanomachine that can reversibly load and release a molecular cargo on binding to a specific antibody. Our strategy takes inspiration from transport proteins, highly evolved machines that are essential to the crucial mechanism of cell transport^33^,^34^. These proteins can load and release a specific molecular cargo through a conformational change mechanism that can be regulated by different inputs^35^. By mimicking this mechanism we designed a DNA-based nanomachine that is able to load a DNA strand in a highly specific and stable fashion and release it only in the presence of a specific antibody.

## Results

### Design of an antibody-powered DNA nanomachine

**Figure 1:**
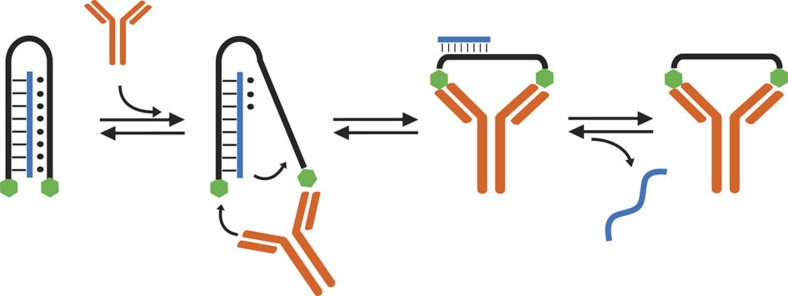
Working principle of antibody-powered DNA-based nanomachine. A DNA strand (black) labelled with two antigens (green hexagons) can load a nucleic acid strand (blue) through a clamp-like triplex-forming mechanism. The binding of a bivalent macromolecule (here an antibody) to the two antigens causes a conformational change that reduces the stability of the triplex complex with the consequent release of the loaded strand.

Our strategy to rationally design an antibody-driven DNA-based nanomachine takes advantage of triplex forming DNA sequences that are designed to recognize a specific DNA strand (blue in Fig. 1) through the formation of a clamp-like structure that involves both Watson–Crick (–) and Hoogsteen (·) interactions (Fig. 1)^36^. This clamp-like structure is conjugated at the two ends with a pair of antigens. Antibody binding to the two antigens on the nanomachine causes a conformational change that induces the triplex-complex opening (see for analogy antibody triggered stem-loop opening)^28^ and energetically disrupts the less stable triplex-forming Hoogsteen interactions (·) thus destabilizing the nanomachine/cargo complex. As the Watson–Crick interactions in such complex are not strong enough to retain the cargo, this latter is released from the nanomachine (Fig. 1).

### Selection of DNA cargo strand

**Figure 2:**
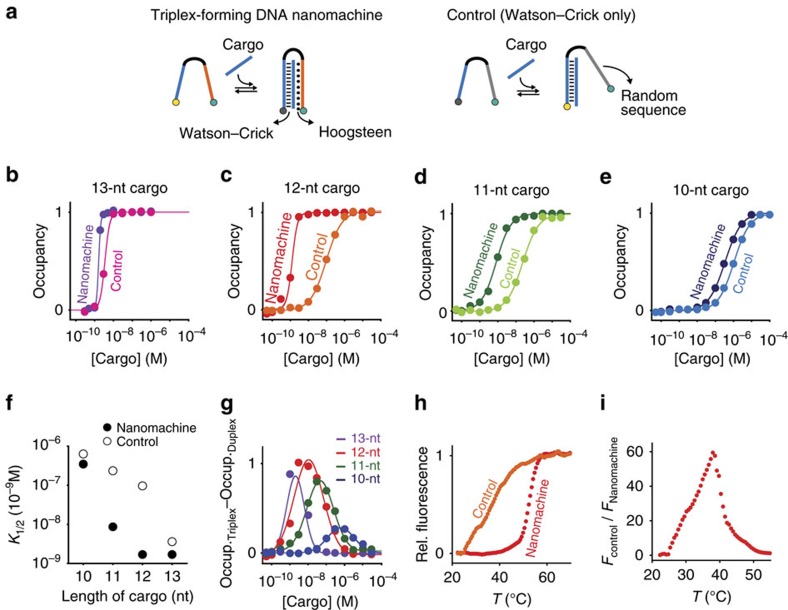
Designing the antibody-powered nanomachine. To find the optimal DNA cargo length to observe the antibody-induced release from the nanomachine, we have compared the binding affinity of a triplex-forming nanomachine with that of a control nanomachine able to only form a duplex complex (**a**) using cargo strands of different length (13 nt (**b**), 12 nt (**c**), 11 nt (**d**) and 10 nt (**e**)). We have observed the strongest difference in affinity (here depicted as the difference of the relative occupancy) between the triplex-forming nanomachine and the control nanomachine with the 12-nt DNA cargo (**f**,**g**). (**h**,**i**) Using the 12-nt DNA cargo, we have also performed melting denaturation experiments showing that, while the triplex complex is stable up to 50 °C (*T*_m_=52.1±0.5 °C), the nanomachine/cargo complex solely based on Watson–Crick interactions (control) shows a melting temperature of 37.0±0.5 °C. The experiments in this figure were performed using a DNA nanomachine (either triplex-forming or control) labelled with a fluorophore/quencher pair (FAM and BHQ-1) so that the binding of the DNA cargo can be easily followed through the decrease or increase, respectively, of the fluorescence signal. The binding curve experiments were performed in 50 mM Na_2_HPO_4_, 150 mM NaCl and 10 mM MgCl_2_ at pH 6.8, 37 °C at a concentration of nanomachine of 3 nM and adding increasing concentrations of cargo strand. Melting curve experiments were performed using the same buffer solution at an equimolar concentration (10 nM) of nanomachine and 12-nt cargo.

Instrumental for our strategy, to observe the antibody-induced DNA cargo release, is the need to find an optimal thermodynamic trade-off that requires to meet the following main conditions. First, a strong difference in stability between the triplex conformation (containing both Watson–Crick and Hoogsteen interactions) and a simple duplex conformation (only Watson–Crick base-pairings). Second, the duplex conformation, under the chosen experimental conditions (for example, temperature and concentration range), should be unstable enough to allow release of the cargo. Finally, the triplex conformation should not be too stable so that bidentate binding to the nanomachine by the antibody would be allowed. To achieve this, we have studied DNA cargos of different length (thus leading to complexes of different stabilities) and tested them with a triplex-forming DNA nanomachine (involving both Watson–Crick and Hoogsteen interactions) and a control DNA nanomachine lacking the triplex forming portion (Fig. 2a). As expected^36^, because of the additional Hoogsteen interactions, for all cargos tested the triplex-forming DNA nanomachine shows a higher affinity (and thus stability) compared to the control nanomachine able to only form a duplex complex (Fig. 2b–e). We find that a 12-nt DNA cargo leads to the strongest difference in affinity between triplex and duplex formation under our experimental conditions (Fig. 2f,g). Using this DNA cargo we show that, while the complex formed with the triplex-forming nanomachine is stable at temperatures below 50 °C (*T*_m_=52.1±0.5 °C), the complex obtained with the control nanomachine (only duplex) is partially unstable at room temperature and leads to an almost complete denaturation at temperatures close to 40 °C (*T*_m_=37.0±0.5 °C) (Fig. 2h,i). In our next experiments we have thus employed a 12-nt DNA strand as our molecular cargo.

### Characterization of antibody-powered DNA nanomachine

**Figure 3:**
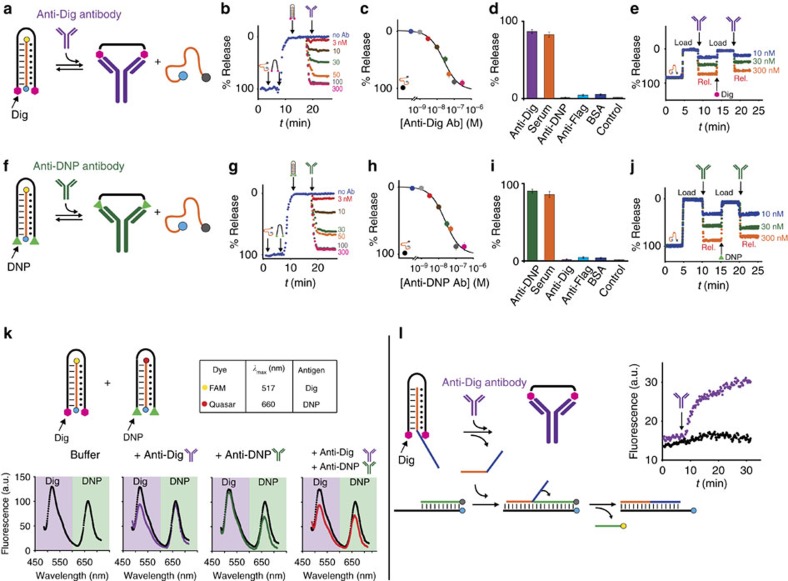
Antibody-powered DNA-based nanomachine. (**a**) We first used digoxigenin (Dig) as antigen and anti-Dig antibodies as molecular triggers of our nanodevices. The nucleic acid cargo strand (orange) is labelled with a fluorophore/quencher pair to easily follow its load/release from the nanomachine. (**b**,**c**) Kinetic profiles show triplex complex formation and subsequent cargo release at different concentrations of anti-Dig antibody. (**d**) The approach is highly specific and works well also in 90% serum (orange bar). (**e**) We can achieve reversible load and release of the molecular cargo by cyclically adding anti-Dig antibody and free Dig in a solution containing both the nanomachine and the cargo strand. (**f**–**j**) Comparable efficiency and results can be achieved using a nanomachine that is labelled with two molecules of DNP at the two ends and thus triggered with anti-DNP antibodies. (**k**) The two nanomachines can orthogonally work in the same solution without crosstalk. (**l**) Moreover, the cargo strand displaced on antibody binding can activate a toehold strand-displacement reaction. The experiments shown in this and in the following figures were performed in 50 mM Na_2_HPO_4_, 150 mM NaCl and 10 mM MgCl_2_ at pH 6.8, 37 °C at an equimolar (50 nM) concentration of nanomachine and cargo unless otherwise noted. Cycles' experiments were performed adding the concentration of antibody indicated in **e**,**j** and a concentration of 300 nM of free Dig or DNP. The experimental values represent mean±s.d. of three separate measurements.

As a first test bed for the optimization of an antibody-powered DNA-based nanomachine we have conjugated the DNA-based triplex-forming nanomachine with two copies of the small-molecule hapten digoxigenin (Dig) at the 5′ and 3′ ends (Fig. 3a) and we have used as triggering input the anti-digoxigenin antibody (anti-Dig) (Fig. 3a). To monitor the release of the cargo we have also labelled the DNA strand cargo with a fluorophore and quencher at the two extremities (Fig. 3a). Because binding of such optically labelled DNA strand cargo to the triplex-forming DNA-based nanomachine causes a conformational stretch that brings the fluorophore faraway from the quencher, we can easily follow its load/release from the DNA-based nanomachine. More specifically, we observe a strong signal increase on loading and a consequent signal decrease when this cargo strand is released from the nanomachine.

Antibody binding to the nanomachine allows to finely modulate the release of the cargo strand. By adding increasing concentrations of anti-Dig antibody to a solution containing the nanomachine/cargo complex, for example, we can release the DNA cargo in a finely controlled manner and achieve an almost complete release (that is, 90±2%) at a 100 nM anti-Dig antibody concentration (Fig. 3b,c). The antibody-induced release is rapid and we achieve equilibration in <60 s. Native polyacrylamide gel electrophoresis (PAGE) experiment (Supplementary Fig. 1) further supports the occurred release of the cargo strand on antibody binding. Of note, under our experimental conditions the nanomachine binds the cargo with high yield: in the absence of antibody more than 94% of cargo is bound to the nanomachine (a value obtained from the affinity constant of the interaction between the 12-base cargo and the Dig-labelled clamp nanomachine, *K*_1/2_=0.20±0.05 nM) and negligible spontaneous leakage of the cargo strand is observed (Supplementary Fig. 2). Moreover, a control experiment using a nanomachine containing only a single Dig hapten shows that binding of the anti-Dig antibody does not lead to any release of the cargo strand (Supplementary Fig. 3). The fitted curve of % cargo release versus antibody concentration (Fig. 3c) appears to be bilinear rather than hyperbolic thus suggesting that we are in the ‘ligand-depletion' regime as the affinity of the antibody for its antigen is well below the 50 nM concentration of the nanomachine employed in our experiment. Consistent with this, the fitted curve gives a *K*_1/2_ (antibody concentration at which the % of cargo release achieved is half the maximum cargo release) of 23±2 nM, which is within error of the 25 nM (half of 50 nM of nanomachine concentration) expected for a stoichiometric 1:1 nanomachine:antibody ratio. To further support this, we have performed antibody-induced cargo release experiments at different concentrations of nanomachine (ranging from 20 to 100 nM) and found that the produced *K*_1/2_ values were always within error of the values expected for a 1:1 stoichiometry (the half of the nanomachine concentration employed; Supplementary Fig. 4).

To confirm the proposed mechanism of our antibody-controlled nanomachine, we have measured the rate of cargo release in presence and absence of the antibody. In presence of antibody (100 nM), the rate of cargo release (*k*_Ab_=0.036 s^−1^) is increased by ∼8-fold compared to that in the absence of antibody (*k*_triplex_=0.0047, s^−1^). Of note, the rate of release in the presence of antibody is similar to the cargo release rate of a duplex control nanomachine (*k*_duplex_=0.058 s^−1^) (Supplementary Fig. 5). Moreover, the antibody-induced cargo release rate is proportional to the concentration of antibody (Supplementary Fig. 6), thus suggesting that antibody binding represents the rate-limiting step of the cargo-release mechanism of the nanomachine. Finally, we also performed binding curves between the labelled cargo strand and the nanomachine in the absence and presence of the specific input antibody (anti-Dig antibody). We found that, as expected, the binding of the antibody to the nanomachine causes a conformational change that affects its ability to form a triplex complex with the cargo strand. As a result, the observed affinity of the reaction leading to the cargo/nanomachine complex gets poorer in the presence of the antibody (Supplementary Fig. 7). These results support the hypothesis that our nanomachine undergoes a conformational change upon binding to the antibody that affects the affinity (and thus release rate) for the cargo strand.

Because the conformational change that causes the DNA cargo release is solely induced by the binding of the specific antibody, this effect is highly specific. We demonstrate that no release of the cargo is observed at saturating concentrations of different non-specific antibodies and proteins (Fig. 3d, Supplementary Fig. 8). A control experiment, employing a DNA-based nanomachine labelled with a single copy of Dig, also provides a confirmation that antibody-induced cargo release requires bivalent binding of the antibody to the nanomachine (Fig. 3d, control). Of note, the binding-induced conformational change that drives cargo release in this nanomachine renders it selective enough to be used in complex sample matrices. The nanomachine, for example, when deployed in 90% bovine blood serum (as a safe and convenient proxy for human samples) shows a cargo release efficiency comparable to that observed in pure buffer (Fig. 3d, orange bar, Supplementary Fig. 9). The nanomachine also works in 100% bovine blood serum although, as expected due to the different pH which affects the stability of the triplex state, with a lower efficiency (Supplementary Fig. 10). The nanomachine is also able to load and release the molecular cargo in a reversible way. We demonstrate this by cyclically adding the specific anti-Dig antibody and the free Dig in a solution containing an equimolar concentration of the nanomachine and DNA cargo (Fig. 3e). The concentration of free Dig (that is, 300 nM) needed to achieve antibody release from the nanomachine and loading of the cargo strand is not as high as expected in the case where a monovalent epitope (free Dig) competes with a bivalent epitope (nanomachine). We note, however, that the presence of the cargo strand strongly supports this competition thus presumably facilitating Dig-induced antibody release from the nanomachine.

The design principle of our antibody-powered DNA nanomachine is highly generalizable and can be easily adapted to other antibodies via the expedient of changing the employed recognition element. To demonstrate this, we have fabricated a second DNA nanomachine construct conjugated with a different antigen (that is, dinitrophenol, DNP) and show that anti-DNP antibodies can trigger the release of a DNA strand cargo with an efficiency, specificity and response time comparable to those observed with the anti-Dig-powered DNA nanomachine (Fig. 3f–j, Supplementary Figs 11 and 12).

Because they specifically respond to their target antibody, different nanomachines can be used orthogonally in the same solution without crosstalk. To demonstrate this, we have employed two different DNA nanomachines responding to anti-Dig and anti-DNP antibodies, respectively (Fig. 3k) in the same solution. Each nanomachine can load and release a DNA strand cargo labelled with a different fluorophore (FAM and Quasar) so that their load/release can be followed separately. The addition of one of the two antibodies in a solution containing both nanomachines causes the release of the specific DNA cargo and only in the presence of both antibodies we observe the release of the two cargos (Fig. 3k).

### Activation of a strand-displacement reaction by antibody binding

The cargo strand released by antibody binding can in principle be used to trigger other chemical or biological functions. In this work we have focused our attention on the toehold strand-displacement reaction, a process through which two DNA strands hybridize with each other displacing one (or more) prehybridized strands. Such reaction has been intensively employed for a wide range of possible applications that include controlled building of complex DNA nanostructures^25^,^37^, control of gene transcription^38^ and biosensing.^39^ To demonstrate toehold strand displacement reaction induced by the antibody released cargo we have designed a 24-nt cargo strand that can trigger a displacement reaction in a preformed target duplex complex. The cargo strand is composed of a 12-nt portion complementary to the nanomachine that also recognizes the toehold binding domain of the preformed target duplex (Fig. 3l, orange portion) and of an additional 12-nt domain (Fig. 3l, blue portion) that acts as invading strand during the displacement reaction. If the cargo is loaded on the nanomachine its binding to the preformed complex cannot occur and thus no displacement reaction is observed. On addition of the antibody the cargo is released and the strand displacement reaction can proceed (Fig. 3l). This effect is specific and no strand displacement is observed on addition of a non-specific antibody (Supplementary Fig. 13).

### Modular antibody-powered DNA nanomachine

**Figure 4:**
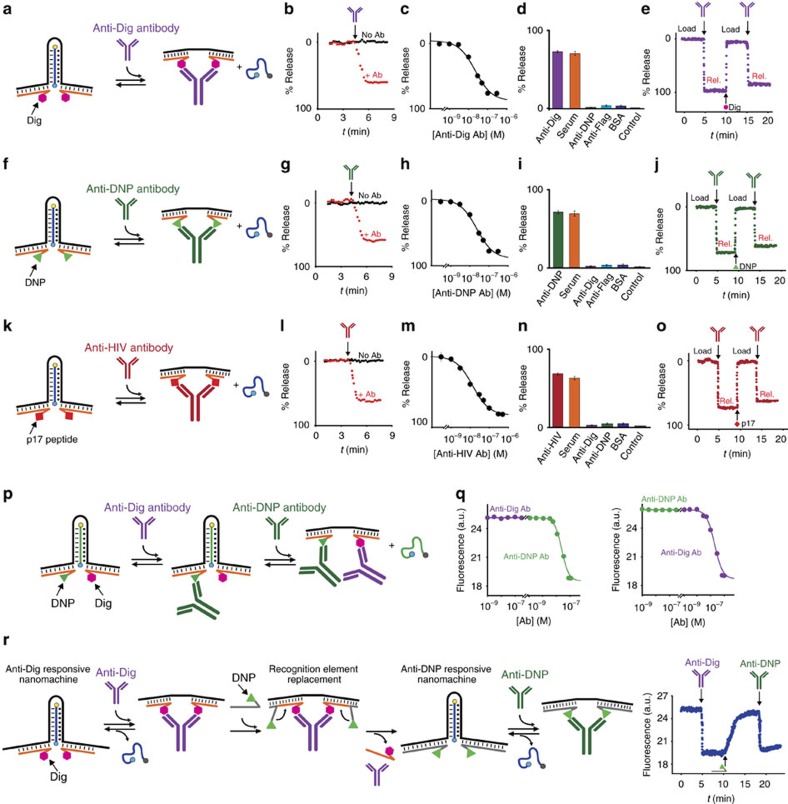
Modular antibody-powered DNA nanomachine. Modular nanomachines employing three different antigens: digoxigenin (**a**–**e**), dinitrophenol (DNP) (**f**–**j**) and a 12-residue epitope (p17 peptide) excised from the HIV-1 matrix protein (**k**–**o**). All these nanomachines are triggered by their specific target antibodies while exhibiting no significant response to high concentrations of the non-specific targets. (**p**) Such modular antibody-powered nanomachine can be adapted to an AND-logic gate that releases its cargo only in the simultaneous presence of two different antibodies. To demonstrate this, we modified a modular nanomachine with the recognition elements Dig and DNP. (**q**) Due to the steric hindrance mechanism that disrupts triplex-forming interactions, we observe the cargo release only in the simultaneous presence of both anti-Dig and anti-DNP antibodies. (**r**) The modular antibody-powered nanomachine also allows to reversibly change the recognition element on the fly via the displacement and substitution of the antigen-conjugated strand (orange and grey). By doing so we can achieve a controlled release of the DNA cargo with two distinct antibodies in the same solution. The experiments reported here were performed in 50 mM Na_2_HPO_4_, 150 mM NaCl and 10 mM MgCl_2_ at pH 6.8, 37 °C at an equimolar (50 nM) concentration of nanomachine, each antigen conjugated strand and cargo. Cycles' experiments were performed adding a concentration of 100 nM of antibody and a concentration of 300 nM of Dig, DNP and p17 peptide. The experimental values represent mean±s.d. of three separate measurements.

A possible limitation of our approach is represented by the need to conjugate the antibody-powered DNA nanomachine with two antigens, a task that could prove challenging from a synthetic point of view. In response to this limitation we have designed a modular version of our nanomachine (Fig. 4a). To do this, we have added to the two ends of the same triplex-forming nanomachine used before two 18-nt DNA tails that can hybridize an antigen-conjugated complementary strand. Such modular DNA nanomachine is thus composed of: (i) a loading module that contains the recognition portion for the DNA strand cargo (black strand in Fig. 4a) and (ii) the triggering module that contains the recognition elements for the specific antibody (orange strand in Fig. 4a). The modular antibody-powered DNA nanomachine designed in this way shows a fast kinetic of release (Fig. 4b) and an efficiency that is comparable to that of the non-modular counterpart. Also in this case we demonstrate cargo release by native PAGE experiments (Supplementary Fig. 14). We show that we can modulate the amount of released cargo by varying the concentration of the triggering antibody (Fig. 4c) and we achieve a high specificity and efficiency even in complex media (that is, 90% serum) (Fig. 4d). Finally, also with the modular nanomachine we observe a reversible load and release activity by cyclically adding the triggering antibody and the free antigen in a solution containing both the nanomachine and the cargo strand (Fig. 4e). The modular nature of this nanomachine allows an easier generalization to other, more complex, recognition elements (and thus triggering antibodies). To demonstrate this, we have used as our recognition element the DNP antigen (Fig. 4f–j) and a short peptide (p17, 12 residues) that is recognized by HIV diagnostic antibodies (Fig. 4k–o). In both cases the effect of the antibody is rapid and specific and we observe efficient load-release of the molecular cargo even in 90% serum (Figs 4i,n). Moreover, the modularity of our approach renders it easy to design a nanomachine that behaves like a AND-logic gate and whose functionality can be triggered only with the concomitant presence of two different antibodies. To demonstrate this, we fabricated a single nanomachine exhibiting two different recognition elements, Dig and DNP (Fig. 4p). The addition of increasing concentrations of either of the targeted antibodies in isolation does not lead to any DNA cargo release (Fig. 4q). As expected, however, cargo release is achieved when the second target antibody is added (Fig. 4q). Finally, the modular nature of our approach also allows to reversibly change the recognition element employed so that the same nanomachine can be triggered by different antibodies with the simple expedient of changing the recognition element. To do this, we have used, in the construction of our nanomachine, slightly shorter DNA strand conjugated with the recognition element (Fig. 4r, orange strand). This allows to displace, using a common DNA strand displacement reaction, the first recognition element conjugated DNA strand and substitute it with a second strand conjugated with a different recognition element. As a proof of principle of this strategy we have first used a nanomachine containing Dig as recognition element (Fig. 4r). In the presence of the anti-Dig antibody the DNA cargo is released as expected (Fig. 4r). The addition of a strand conjugated with DNP (Fig. 4r, grey strand) allows to displace the Dig-conjugated strand and the anti-Dig antibody and to restore cargo loading. Such nanomachine can now be triggered in the presence of anti-DNP antibody causing a new release of the DNA cargo (Fig. 4r).

## Discussion

Despite the fact that several DNA-based platforms have been demonstrated for the detection of specific antibodies^28^,^29^,^30^, only few examples have been reported to date where a certain function of DNA-based nanomachine can be controlled by these important biomolecules^31^,^32^. In response to this consideration and taking inspiration from naturally occurring transport factors, proteins that bind a molecular cargo and release it only on an input-induced conformational change^33^,^34^,^35^, here we have designed a new class of DNA-based nanomachines that can load and release a molecular cargo on the binding of a specific target antibody. The system we propose here is highly versatile and in principle, generalizable to any antibody for which an antigen can be attached to a DNA-anchoring strand. In support of this claim, we have demonstrated here that our approach can be extended to three different triggering antibodies and the effect can be specific and selective enough even in complex media (90% serum). We have also demonstrated that our nanomachine can reversibly load and release the cargo on cyclic addition of the specific antibody and of the free antigen and that the modularity of our approach allows to design nanomachines that can respond to different antibodies in an orthogonal way or whose recognition module can be substituted on-the-fly on need. And while many examples have been reported where the release of DNA strands can be controlled by several molecular cues (that is, pH^40^, proteins^41^ and so on), the possibility to use antibodies as triggering input to release a specific DNA strand might open new routes in the field of DNA nanotechnology. For example, because antibodies represent a wide class of clinical and diagnostic markers, the antibody-powered DNA nanomachines we have developed here may be useful in a range of applications, including point-of-care diagnostics, controlled drug-release and *in vivo* imaging.

Finally, we have demonstrated that such strategy can be successfully used to activate a toehold strand displacement reaction on antibody binding. Because the toehold DNA strand displacement process has been used to assemble dynamic and static DNA-based nanostructures^25^,^37^, it would be in principle straightforward to rationally design a DNA self-assembly process that would allow to assemble or disassemble DNA nanostructures using potentially any antibody as the triggering molecular input. This would allow to design and build novel DNA nanostructures whose diagnostic or drug-delivery function could be triggered using specific diagnostic or clinically relevant antibodies.

## Methods

### Chemicals

Sheep polyclonal anti-Dig antibodies were purchased from Roche Diagnostic Corporation (Germany), mouse monoclonal anti-DNP antibodies were purchased from Sigma-Aldrich, USA, murine monoclonal anti-HIV antibodies were purchased from Zeptometrix Corporation, USA, rat monoclonal anti-FLAG antibodies were purchased from Novus Biologicals, UK. All the antibodies were aliquoted and stored at 4 °C for immediate use or at −20 °C for long-term storage. Bovine serum albumin (A4503), fetal bovine serum (F0804), digoxigenin (D9026) and 2,4-dinitrophenol (D198501) were purchased from Sigma-Aldrich (Italy).

### Oligonucleotides and DNA-based nanomachines

High-performance liquid chromatography-purified oligonucleotides were purchased from IBA (Gottingen, Germany) or Biosearch Technologies (Risskov, Denmark). The DNA strand cargos or the DNA nanomachines were modified with FAM (5-carboxyfluorescein) or Quasar670 and BHQ-1 (black hole quencher 1) or BHQ-2 (black hole quencher 2). The sequences and modification schemes are as follows:

Triplex-forming DNA-based nanomachine: 5′-(FAM) TCTCTCCTTTCTCCTGTTTCTCCTCTTTCCTCTCT (BHQ1)-3′

Duplex-forming DNA-based nanomachine (control): 5′-(FAM) TCTCTCCTTTCTCCTGTTTCTTTTTTTTTTTTTTT (BHQ1)-3′

DNA cargo 13 nt: 5′-GAGAAAGGAGAGA-3′

DNA cargo 12 nt: 5′-AGAAAGGAGAGA-3′

DNA cargo 11 nt: 5′-GAAAGGAGAGA-3′

DNA cargo 10 nt: 5′-AAAGGAGAGA-3′

Anti-Dig-powered DNA-based nanomachine: 5′-(Dig) TCTCTCCTTTCTGTTTCTCTTTCCTCTCT (Dig)-3′

Anti-DNP powered DNA-based nanomachine: 5′-(DNP) TCTCTCCTTTCTGTTTCTCTTTCCTCTCT (DNP)-3′

DNA cargo 12 nt: 5′-(FAM) AGAAAGGAGAGA (BHQ1)-3′

DNA cargo 11 nt: 5′-(FAM) GAAAGGAGAGA (BHQ1)-3′

DNA cargo 10 nt: 5′-(FAM) AAAGGAGAGA (BHQ1)-3′

Anti-Dig-powered DNA-based nanomachine (single-labelled control): 5′-(Dig) TCTCTCCTTTCTGTTTCTCTTTCCTCTCT-3′

Modular DNA-based nanomachine: 5′-*ATGGCATTAACCTTGCT*TCTCTCCTTTCTGTTCTCTTTCCTCTCT**AGGTTCATCATCAACTAG**-3′

Here the portions in italic represents tail 1, where the first antigen-conjugated strand hybridizes. The portions in bold represents tail 2, where the second antigen-conjugated strand hybridizes.

We also designed a nanomachine containing a frame inversion at the junction of one of its two tails with the following sequence:

Modular DNA-based nanomachine (frame inversion): 5′-*CAAGAATAAAACGCCACTGT*TCTCTCCTTTCTGTTCTCTTTCCTCTCT-3′–3′-*GTCACCGCAAAATAAGAACA*-5′

Here the portions in italic represent the two tails and have same sequence but are oriented head-to-head due to the indicated (3–3′) frame inversion. This nanomachine allows to use a single-recognition element-conjugated strand to bind both tails thus lowering the production costs.

Recognition-element-conjugated oligonucleotides (DNA/PNA as denoted below) were used as received from the appropriate vendors. The sequences and modification schemes are as follows:

Dig-labelled strand (tail 1): 5′-(Dig) AGCAAGGTTAATGCCAT-3′

Dig-labelled strand (tail 2): 5′-CTAGTTGATGATGAACCT (Dig)-3′

DNP-labelled strand (tail 1): 5′-(DNP) AGCAAGGTTAATGCCAT-3′

DNP-labelled strand (tail 2): 5′-CTAGTTGATGATGAACCT (DNP)-3′

In the sequences above, Dig was introduced onto the DNA via EDC/NHS coupling to an amine attached via a 5-carbon linker on the 5′ end or on the 3′ end. DNP was attached via a triethylene glycol (TEG) spacer arm on either the 5′ or the 3′ terminus of the appropriate oligonucleotide.

When using p17 as the recognition element we have employed a p17-PNA chimera as this is more convenient to fabricate than the equivalent DNA-polypeptide chimera. The p17-conjugated sequence we employed is as follows:

p17-labelled strand: N_term_−*ELDRWEKIRLRP*−CAGTGGCGTTTTATTCT-C_term_

The sequences in italics represent the amino-acid (standard one-letter code) sequence of the polypeptide antigen. This peptide-labelled strand was used with the modular DNA-based nanomachine containing the frame inversion (see above).

### Antibody-induced toehold strand-displacement reaction strands

To activate toehold strand-displacement reaction by the released cargo (Fig. 3l) we have used the anti-Dig-powered DNA-based nanomachine (see sequence above) and the following sequence as cargo strand:

Invanding cargo strand: 5′-**AGAAAGGAGAGA***AAGGAAAGAGGA*-3

In this sequence the portion in bold (12 nucleotides) represents the domain recognized by the DNA-nanomachine while the portion in italics represents the strand-invading domain.

The two strands forming the target duplex used for this experiment are labelled with Quasar570 and Quasar 670 and have the following sequences:

Strand 1: 5′-AAGGAAAGAGGAAGAAAA (Quasar570)-3′

Strand 2: 5′-(Quasar670) TTTTCTTCCTCTTTCCTTTCTCTCCTTTCT-3′

### Substitution and displacement strands

The following sequences and strands were used to reversibly change the recognition element on the fly via the displacement and substitution of the antigen-conjugated strand (Fig. 4r):

DNA-based nanomachine: 5′-CTTCGAATGGCATTAACCTTGCTTCTCTCCTTTCTGTTCTCTTTCCTCTCTAGGTTCATCATCAACTAGCTTTCT-3′

Dig-labelled strand (tail 1): 5′-(Dig) AGCAAGGTTAATGCCAT-3′

Dig-labelled strand (tail 2): 5′-CTAGTTGATGATGAACCT (Dig)-3′

DNP-labelled displacement strand (tail 1): 5′-(DNP) AGCAAGGTTAATGCCATTCGAAG-3′

DNP-labelled displacement strand (tail 2): 5′-AGAAAGCTAGTTGATGATGAACCT (DNP)-3′

### Fluorescent experiments

Fluorescent experiments were conducted at pH 6.8 in 50 mM Na_2_HPO_4_ buffer, 150 mM NaCl, 10 mM MgCl_2_ at 37 °C in a 100 μl cuvette (total volume of the solution 100 μl). Equilibrium fluorescence measurements were obtained using a Cary Eclipse Fluorimeter respectively with excitation at 490 (±5) nm (for DNA strands labelled with FAM) and acquisition at 517 (±5) nm or with excitation at 647 (±5) nm (for DNA strands labelled with Quasar670) and acquisition at 655 (±5) nm. Melting curves were obtained by preparing a 100 μl solution containing 10 nM of DNA-based nanomachine and 10 nM of DNA cargo strand and waiting 10 min for reaction before temperature ramping. Temperature was ramped between 20 and 70 °C at 1 °C min^−1^. Data were normalized on a scale from 0.01 (set as background signal) to 1. In Fig. 2h, we have subtracted the normalized values of the control nanomachine to 1.01 to better compare triplex-forming nanomachine (signal-on) and control nanomachine (signal-off) results. Binding curves were obtained by preparing a 100 μl solution containing 50 nM of DNA-based nanomachine and 50 nM of DNA cargo strand and by sequentially adding increasing concentrations of the target antibody. Binding curve of the modular DNA-based nanomachines were obtained by preparing a 100 μl solution containing 50 nM of DNA-based nanomachine, 50 nM of DNA cargo strand and 50 nM of each antigen-conjugated strand and by sequentially increasing the concentration of the target antibody. For each concentration, the fluorescence signal was recorded every 10 min until it reached equilibrium. For experiments performed in serum we mixed the serum (90%) with a 10 × buffer (10%) (500 mM Na_2_HPO_4_, 1.5 M NaCl and 100 mM MgCl_2_ at pH 6.8) so that the final ionic strength of the solution is similar to that used in previous experiments (50 mM Na_2_HPO_4_, 150 mM NaCl and 10 mM MgCl_2_, pH 6.8) at 37 °C. For strand displacement experiments, we have used a concentration of target duplex complex of 10 nM and followed the signal of the released strand labelled with Quasar570 with excitation at 540 (±5) nm and acquisition at 566 (±5) nm.

For the binding curves, the observed fluorescence in the presence of different concentrations of antibody, *F*_[antibody]_, was fitted using the following four parameter logistic equation (equation (1)):^42^


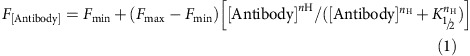


where, *F*_min_ and *F*_max_ are the minimum and maximum fluorescence values, *K*_1/2_ is the equilibrium antibody concentration at half-maximum signal, *n*_H_ is the Hill coefficient and [Antibody] is the concentration of the specific antibody added. This model is not necessarily physically relevant, but it does a good (empirical) job of fitting effectively bi-linear binding curves such as those we obtain for most of our nanomachines, providing a convenient and accurate means of estimating *K*_1/2_. The signals obtained in Fig. 2 with the triplex-forming and control nanomachine have been normalized on a 0–1 scale to allow for more ready interpretation of the results. More specifically, the relative occupancy (defined as the fraction of nanomachine bound to the cargo) was plotted against the cargo concentration. To obtain the relative occupancy, we considered the maximum signal of the triplex forming nanomachine as the signal of the unbound nanomachine (occupancy=0) while the minimum signal was considered as the signal of the completely bound nanomachine (occupancy=1). Conversely, for the duplex-control nanomachine (Fig. 2a) we considered the minimum signal as the signal of the unbound nanomachine (occupancy=0) and the maximum signal as the signal of the completely bound nanomachine (occupancy=1). In the other figures the % of cargo release was plotted against antibody concentration. In this case, the maximum cargo release (100%) was considered as the signal corresponding to the free cargo (in absence of nanomachine), while the minimum cargo release (0%) was considered as the signal of the completely bound cargo (in the presence of a saturating amount of nanomachine).

### Native PAGE experiments

Native polyacrylamide gel (18%) was first incubated with running buffer (1 × TAE solution, pH 6.5) for 1 h at 37 °C. A volume of 30 μl of each DNA sample was mixed with 3.5 μl of glycerol, and then the mixture was added into the gel for electrophoresis. The native PAGE was carried out in a Mini-PROTEAN Tetra cell electrophoresis unit (Bio-Rad) at 37 °C, using 1 × TAE buffer at pH 6.5 and at a constant voltage of 50 V for 2 h 30 min (using Bio-Rad PowerPac Basic power supply). After 30 min of staining in 1 × SYBR gold (Invitrogen) (dissolved in a 1 × TAE buffer at pH 8.0), the gel was scanned by a Gel Doc XR+ system (Bio-Rad). In these experiments we used the following modified cargo strand containing the usual recognition domain and a 16-nt hairpin tail (bold below) that allow dye intercalation:

Cargo strand Gel: 5′-**CTGCGTTTCGCAGTTT**AGAAAGGAGAGA-3′

### Data availability

Data supporting the findings of this study are available within the article (and its Supplementary Information files) and from the corresponding author on reasonable request.

## Additional information

**How to cite this article:** Ranallo, S. *et al*. Antibody-powered nucleic acid release using a DNA-based nanomachine. *Nat. Commun.* **8,** 15150 doi: 10.1038/ncomms15150 (2017).

**Publisher's note:** Springer Nature remains neutral with regard to jurisdictional claims in published maps and institutional affiliations.

## Supplementary Material

Supplementary Information Supplementary Figures.
