# Supplementary material for: Antibody-powered nucleic acid release using a DNA-based nanomachine
Source: Nat Commun. 2017 May 8;8:15150. doi: 10.1038/ncomms15150 (PMC5424144; doi:10.1038/ncomms15150)
Supplement: Supplementary Information — Supplementary Figures. [file ncomms15150-s1.pdf]

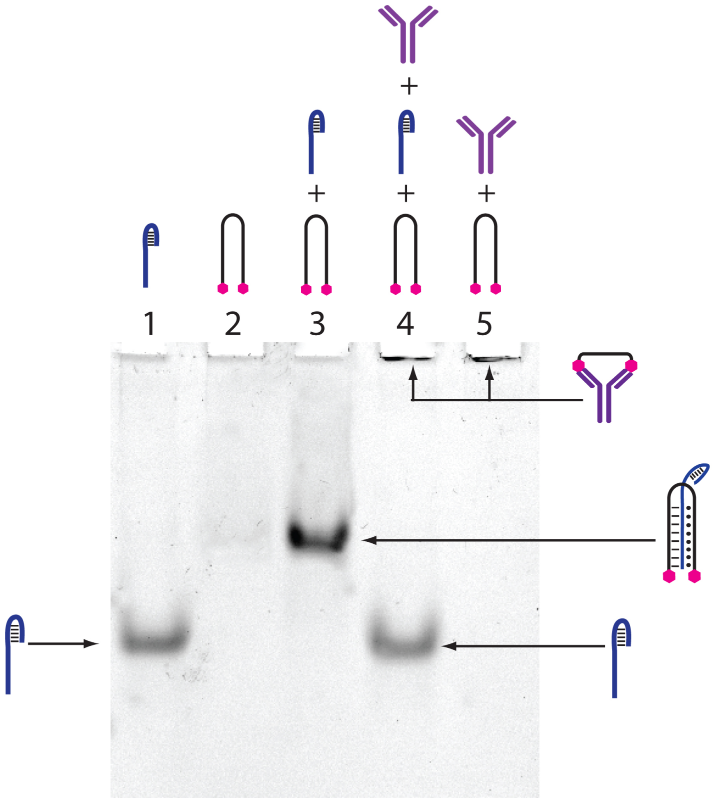

Supplementary Figure 1. Antibody-induced cargo release studied by native PAGE. A clear band corresponding to the cargo strand (lane 1) is visible. Because SYBR Gold is less sensitive to single stranded DNA, the Dig-labeled nanomachine (Figure 3a) does not show any visible band under these conditions (lane 2). Using a solution containing both the nanomachine and the cargo strand a new band, corresponding to the nanomachine/cargo complex appears (lane 3). In the presence of the antibody, it is possible to observe the band corresponding to the cargo strand while that of the nanomachine/cargo complex is not visible (lane 4). The band of the nanomachine/antibody complex, due to the high molecular weight, shows, as expected, a very limited mobility (lane 4 and 5). Here native PAGE containing 18% polyacrylamide (29:1 acrylamide/bisacrylamide) was run on a Mini-PROTEAN Tetra cell electrophoresis unit (Bio-Rad) at 37°C (50 V) for 2h 30min in 1x TAE buffer, pH 6.5. After electrophoresis, the gels were stained with 1x SYBR gold and scanned with a Gel Doc XR+ (Bio-Rad).

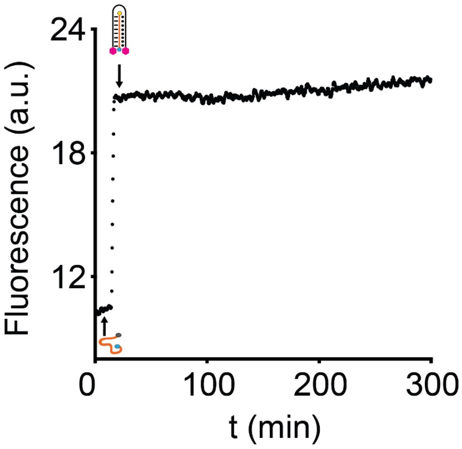

Supplementary Figure 2. Nanomachine/cargo complex reaction. We have continuously measured the fluorescence signal of the Dig-labeled nanomachine bound to the cargo strand for a total time of 300 minutes. No significant signal decrease was observed thus demonstrating that the cargo is efficiently retained by the nanomachine (leakage of the labeled cargo will result in a decrease of the fluorescence signal). Here kinetic fluorescent experiments were performed under the same conditions employed in Figure 3 (50 mM Na_2HPO_4, 150 mM NaCl and 10 mM $MgCl_2$ at pH 6.8, 37°C at an equimolar (50 nM) concentration of nanomachine and cargo).

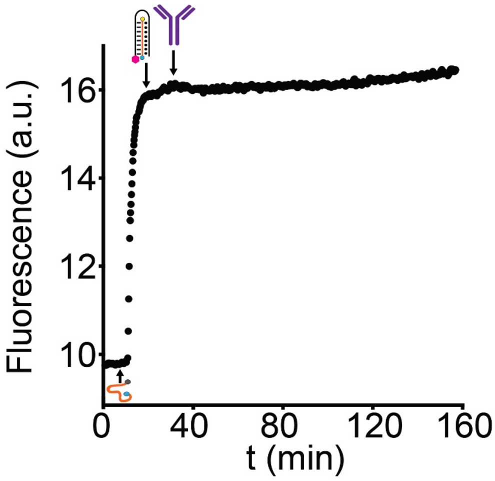

Supplementary Figure 3. Effect of antibody binding to the stability of nanomachine/cargo complex. To demonstrate that the binding of the antibody to a single antigen in the nanomachine/cargo complex does not affect its stability, we have continuously measured the fluorescence signal of a nanomachine containing only a single Digoxigenin hapten bound to the cargo for a total time of 160 minutes. By adding the Anti-Dig antibody at saturating concentration (i.e. 300 nM) no significant signal decrease was observed thus demonstrating that the complex is stable and no leakage of the cargo occurs upon antibody binding to the Digoxigenin hapten (leakage of the labeled cargo will result in a decrease of the fluorescence signal). Here kinetic fluorescent experiments were performed under the same conditions employed in Figure 3 (50 mM Na_2HPO_4, 150 mM NaCl and 10 mM $MgCl_2$ at pH 6.8, 37°C at an equimolar (50 nM) concentration of nanomachine and cargo).

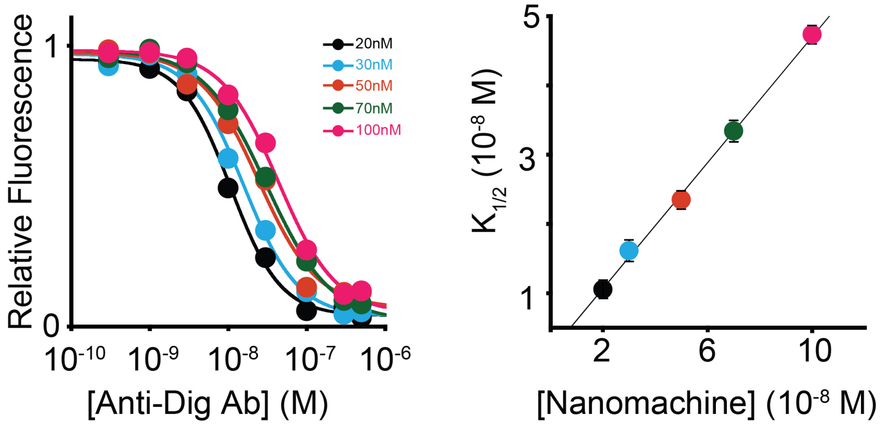

Supplementary Figure 4. $K_{1/2}$ values (the concentration of antibody at which the % cargo release achieved is half the maximum cargo release) change with varying nanomachine's concentration in precisely the manner expected for a 1:1 binding stoichiometry, thus supporting the proposed mechanism. The experiments shown here were performed in 50 mM Na_2HPO_4, 150 mM NaCl and 10 mM $MgCl_2$ at pH 6.8, 37°C at an equimolar concentration of nanomachine and cargo. The experimental values represent mean±s.d. of three separate measurements.

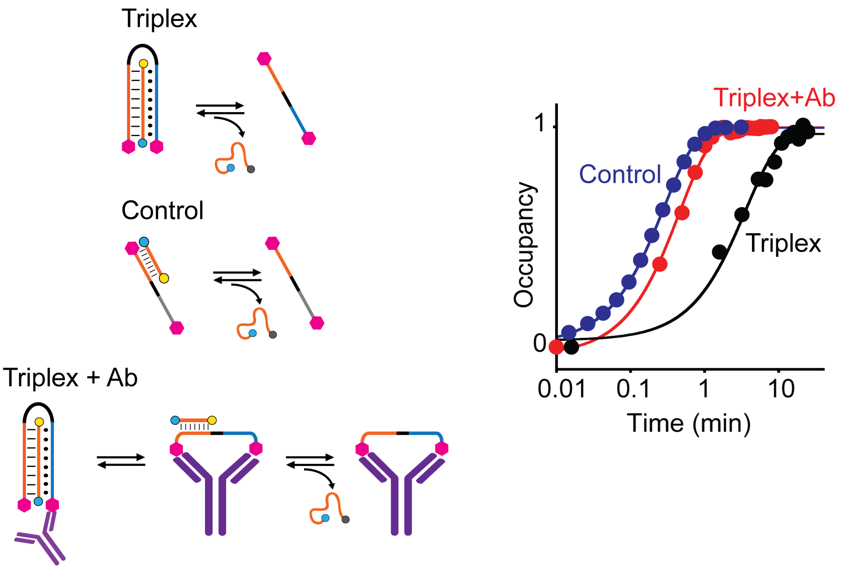

Supplementary Figure 5. Antibody-induced cargo release rate. The cargo release rate in presence of antibody (100 nM) (k_{Ab} = 0.036 s^{-1}) is ~8-fold higher than that observed in the absence of antibody ($k_{triplex}$ = 0.0047 s^{-1}) and similar to the cargo release rate of a duplex "control" nanomachine (k_{duplex} = 0.058 s^{-1}). The cargo release rate from the nanomachine in absence of the antibody ($k_{triplex}$) was measured by performing a kinetic experiment where a saturating amount of unlabeled cargo (5 μM) was added to a solution containing the nanomachine previously bound to the labeled cargo (at equimolar concentration, 50 nM). As the nanomachine spontaneously releases its labeled cargo (leading to a decrease in fluorescence) it is rapidly reloaded (reloading rate is not limiting due to the high concentration of unlabeled cargo) with the unlabeled cargo. The kinetic of this reaction provides an accurate estimation of the rate of cargo release from the nanomachine in the absence of antibody. Similarly, cargo release rate from the duplex control was measured by performing a kinetic experiment where a saturating amount of unlabeled cargo (5 μM) was added to a solution containing the control nanomachine previously bound to the labeled cargo (at equimolar concentration, 50 nM). In all cases the kinetic profiles were fitted to a single-exponential function to obtain the first-order kinetic constants. See Supplementary Figure 6 for experimental details to obtain rate values of antibody-induced cargo release.

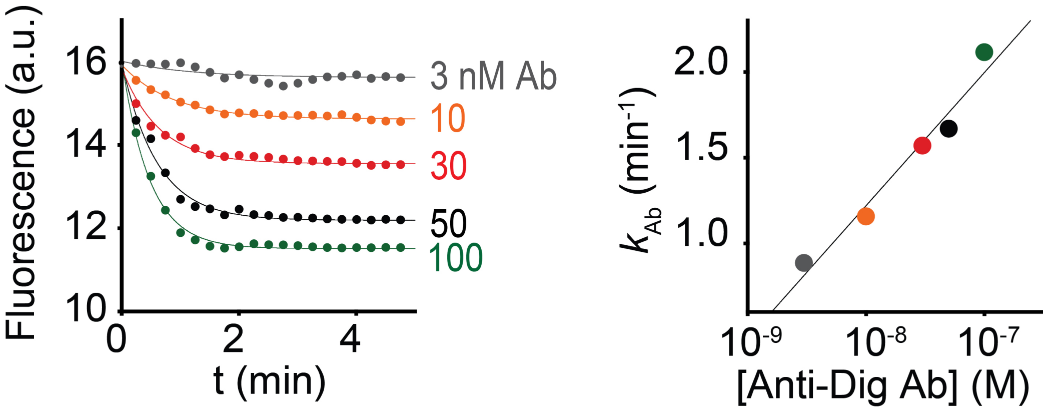

Supplementary Figure 6. Antibody-induced cargo release rate. Antibody-induced cargo release experiments at different antibody concentrations show that the observed rate of cargo release is proportional to the concentration of antibody thus suggesting that antibody binding represents the rate limiting step of the cargo-release mechanism of the nanomachine. The experiments shown in this figure were performed in 50 mM Na_2HPO_4, 150 mM NaCl and 10 mM $MgCl_2$ at pH 6.8, 37°C at an equimolar (50 nM) concentration of nanomachine and cargo and adding the indicated concentration of antibody. The kinetic profiles were fitted to a single-exponential function to obtain the first-order kinetic constants.

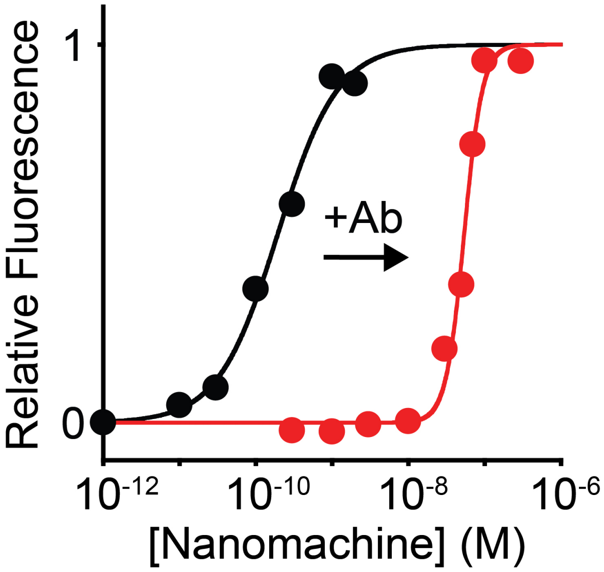

Supplementary Figure 7. The binding curves obtained with the labeled cargo strand and at increasing concentrations of the nanomachine containing Digoxigenin (Figure 3a) in the absence (black curve) and presence (red curve) of a saturating amount of Anti-Dig antibody (i.e. 300 nM) show that, as expected, the binding of the antibody to the nanomachine affects its affinity for the cargo strand. The experiments shown here were performed in 50 mM Na_2HPO_4, 150 mM NaCl and 10 mM $MgCl_2$ at pH 6.8, 37°C at a fixed concentration of labeled cargo strand (0.4 nM) and adding increasing concentrations of digoxigenin-nanomachine (Figure 3a).

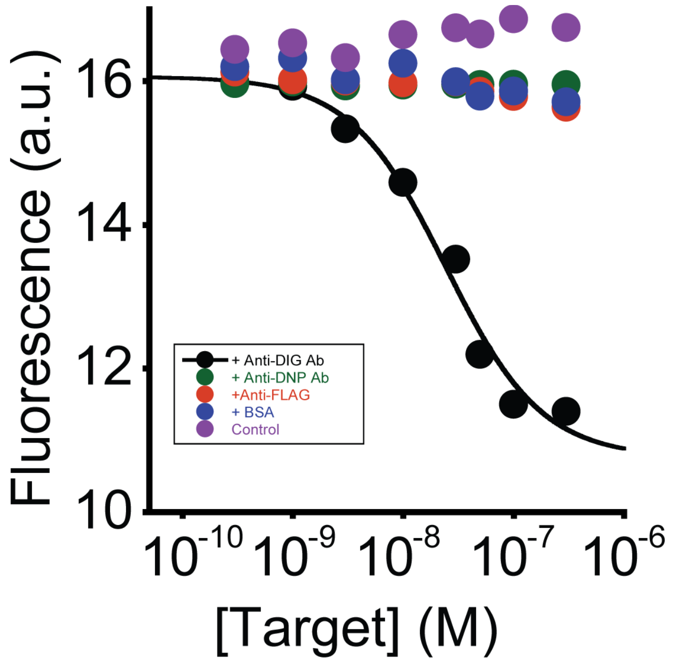

Supplementary Figure 8. Anti-Digoxigenin antibody powered nanomachine. The DNA cargo strand is released upon the binding of the specific anti-Digoxigenin antibody to the two antigens (Digoxigenin) conjugated to the DNA-based nanomachine (black curve). The mechanism is highly specific and we do not observe any significant cross-reactivity with other antibodies and proteins even at saturating concentrations (Anti-DNP antibody, green dots; Anti-Flag antibody, red dots; BSA, blue dots). Reported is also the binding curve obtained testing a control DNA nanomachine (conjugated with only one Dig molecule) with Anti-Dig antibody (purple dots). Here binding curves were performed in 50 mM Na_2HPO_4, 150 mM NaCl and 10mM $MgCl_2$ at pH 6.8, 37 °C at an equimolar (50 nM) concentration of nanomachine and cargo adding increasing concentrations of the indicated antibodies or proteins. The experimental values represent mean±s.d. of three separate measurements.

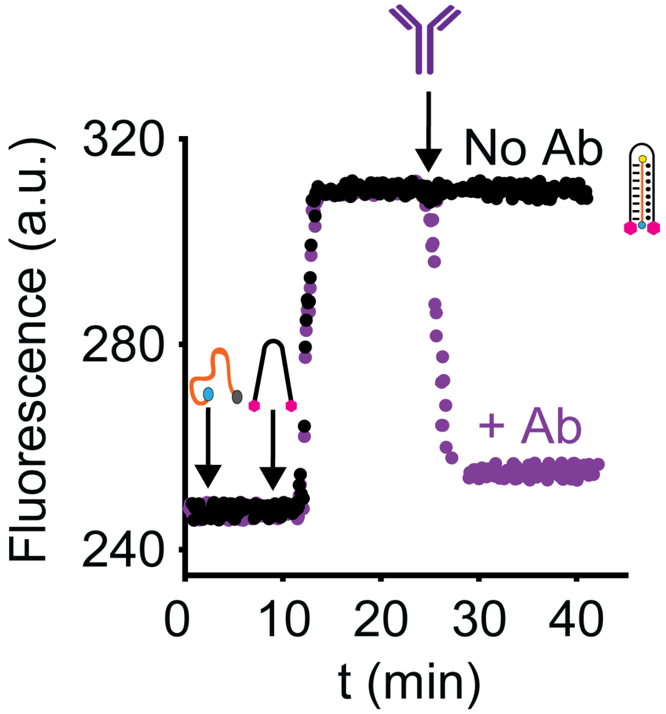

Supplementary Figure 9. Our Anti-Dig antibody powered nanomachine is rapid and specific enough to work in complex clinical samples. Here is shown the kinetic profile obtained in a 90% bovine serum solution containing the optical-labeled molecular cargo (50 nM) upon the addition of the Anti-Dig antibody powered nanomachine (50 nM) and subsequent addition of the specific Anti-dig antibody (300nM). Here the experiments were performed in a solution of 90% bovine serum diluted in buffer (500 mM Na_2HPO_4, 1.5 M NaCl and 100mM $MgCl_2$ at pH 6.8) at 37 °C.

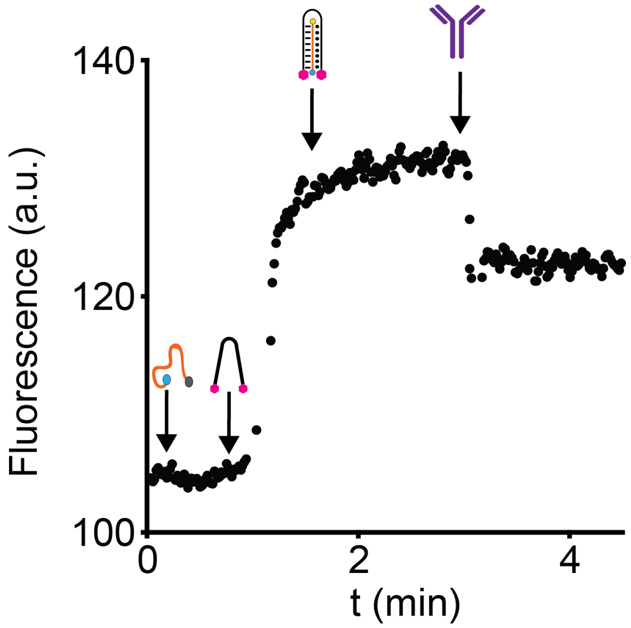

Supplementary Figure 10. Our Anti-Dig antibody powered nanomachine also works, although with a lower efficiency, in 100% blood serum. Here is shown the kinetic profile obtained in a 100% bovine serum solution containing the optical-labeled molecular cargo (50 nM) upon the addition of the Anti-Dig antibody powered nanomachine (50 nM) and subsequent addition of the specific Anti-dig antibody (300nM).

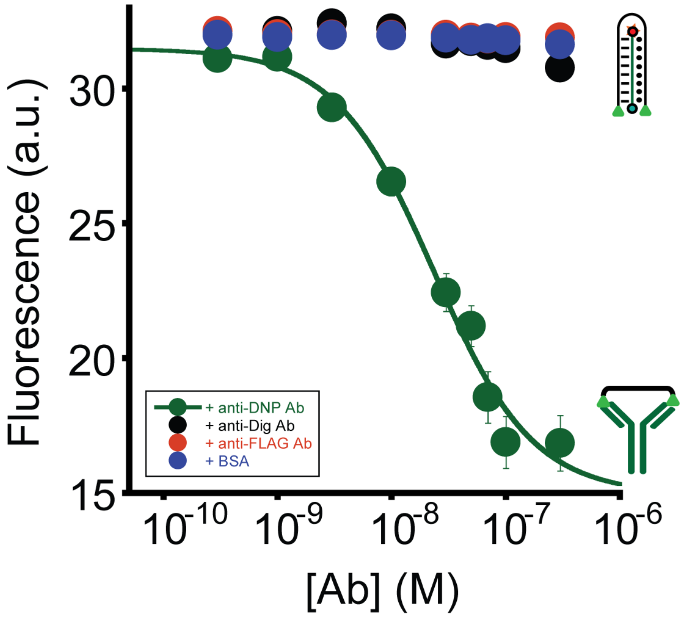

Supplementary Figure 11. Anti-DNP antibody powered nanomachine. The DNA cargo strand is released upon the binding of the specific anti-DNP antibody to the two antigens (DNP) conjugated to the DNA-based nanomachine (green curve). The mechanism is highly specific and we do not observe any significant cross-reactivity with other antibodies and proteins even at saturating concentrations (Anti-Dig antibody, black dots; Anti-Flag antibody, red dots; BSA, blue dots). Here binding curves were performed in 50 mM Na_2HPO_4, 150 mM NaCl and 10mM $MgCl_2$ at pH 6.8, 37 °C at an equimolar (50 nM) concentration of nanomachine and cargo adding increasing concentrations of the indicated antibodies or proteins. The experimental values represent mean±s.d. of three separate measurements.

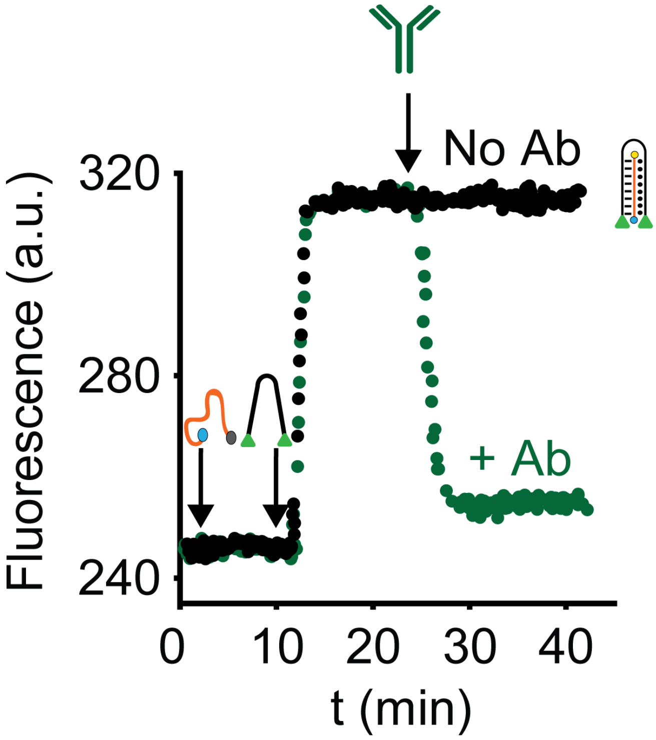

Supplementary Figure 12. Our Anti-DNP antibody powered nanomachine is rapid and specific enough to work in complex clinical samples. Here is shown the kinetic profile obtained in a 90% bovine serum solution containing the optical-labeled molecular cargo (50 nM) upon the addition of the Anti-DNP antibody powered nanomachine (50 nM) and subsequent addition of the specific Anti-DNP antibody (300nM). Here the experiments were performed in a solution of 90% bovine serum diluted in buffer (500 mM Na_2HPO_4, 1.5 M NaCl and 100mM $MgCl_2$ at pH 6.8) at 37 °C.

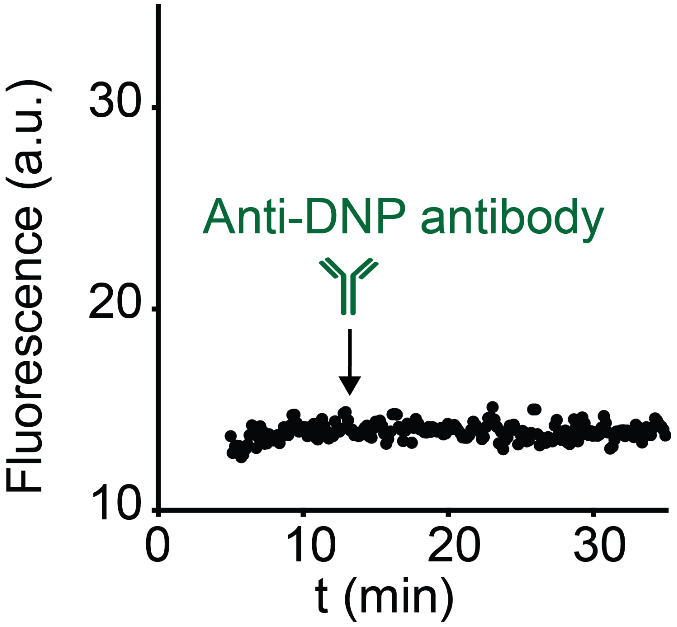

Supplementary Figure 13. Specificity of toehold-strand displacement reaction activated by antibody binding. By adding to the same system shown in Figure 3l a saturating concentration (i.e. 300 nM) of a non-specific antibody (Anti-DNP antibody) we do not observe any activation of the toehold strand displacement reaction. This further demonstrates the specificity of the toehold strand displacement reaction activated by the cargo strand released by the specific antibody (Figure 3l). See methods section for details on the experimental procedure.

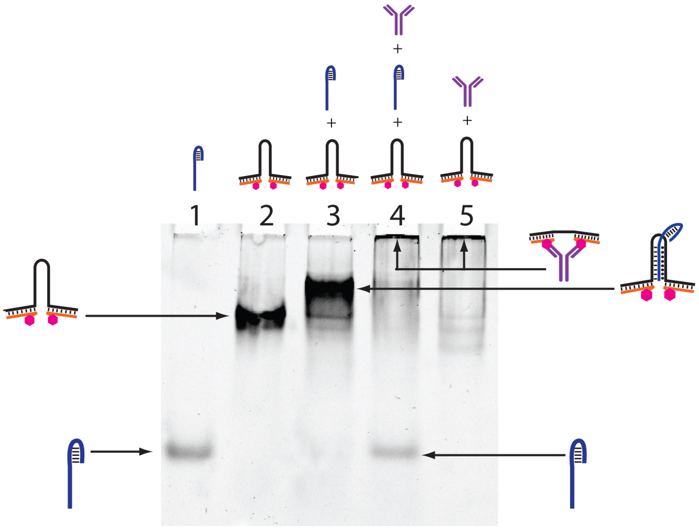

Supplementary Figure 14. Antibody-induced cargo release from the modular nanomachine studied by native PAGE. Two clear bands corresponding to the cargo strand (lane 1) and the Dig-labeled modular nanomachine (Figure 4a) (lane 2) are visible. Using a solution containing both the nanomachine and the cargo strand a new band, corresponding to the nanomachine/cargo complex appears (lane 3). In the presence of the antibody, it is possible to observe the band corresponding to the cargo strand while that of the nanomachine/cargo complex is not visible (lane 4). The band of the nanomachine/antibody complex, due to the high molecular weight, shows, as expected, a very limited mobility (lane 4 and 5). Here native PAGE containing 18% polyacrylamide (29:1 acrylamide/bisacrylamide) was run on a Mini-PROTEAN Tetra cell electrophoresis unit (Bio-Rad) at 37°C (50 V) for 2h 30min. in 1x TAE buffer, pH 6.5. After electrophoresis, the gels were stained with 1x SYBR gold and scanned with a Gel Doc XR+ (Bio-Rad).
